# Post Approval Experience with Caplacizumab for Acquired Thrombotic Thrombocytopenic Purpura at a Single Institution

**DOI:** 10.3390/jcm10153418

**Published:** 2021-07-31

**Authors:** Constantine N. Logothetis, Ankita Patel, Jennifer Eatrides, Michael Jaglal, Mintallah Haider, Nathan Visweshwar, Damian A. Laber

**Affiliations:** 1 Division of Hematology/Oncology, Department of Internal Medicine, Morsani College of Medicine, University of South Florida, Tampa, FL 33612, USA; ankita.patel@moffitt.org (A.P.); jennifer.eatrides@moffitt.org (J.E.); michael.jaglal@moffitt.org (M.J.); mintallah.haider@moffitt.org (M.H.); nviswesh@usf.edu (N.V.); damian.laber@moffitt.org (D.A.L.); 2 Section of Satellite and Community Oncology, H. Lee Moffitt Cancer Center and Research Institute, Tampa, FL 33612, USA

**Keywords:** caplacizumab, aTTP, treatment, Acquired Thrombotic Thrombocytopenic Purpura

## Abstract

Caplacizumab prevents platelet adhesion and has been approved for acquired thrombotic thrombocytopenic purpura (aTTP). This study was retrospective, including all patients diagnosed with aTTP and treated with caplacizumab since commercial availability in 2019 until 28 February 2021 at a single academic hospital with no exclusion criteria. Results used definitions for outcomes in aTTP from the International Working Group Consensus. Ten patients with aTTP received caplacizumab. The median age was 52 years. Six (60%) patients had refractory aTTP while 4 (40%) had newly diagnosed aTTP. The median laboratory values prior to therapy demonstrated: platelet count (PC) 29/uL, LDH 518 U/L (182–1850), ADAMTS13 activity 3% and ADAMTS13 inhibitor 1.4 BU. Everyone received glucocorticoids, rituximab, therapeutic plasma exchange (TPE) and caplacizumab. The median number of TPE was 12 days. Caplacizumab was started at a median of 5 days after the first TPE and the median treatment duration was 31 days. Normalization of PC, LDH and ADAMTS13 activity in days were 5, 3.5, and 32.5, respectively. Six (60%) patients achieved complete response, 3 (30%) had refractory disease and 1 (10%) had relapsed aTTP. No subject suffered abnormal bleeding, or thrombotic event. There were no deaths. Caplacizumab with TPE, glucocorticoids and rituximab was a safe and effective therapy for aTTP.

## 1. Introduction

Acquired thrombotic thrombocytopenic purpura (aTTP) is a thrombotic microangiopathy (TMA) syndrome characterized clinically by the presence of microangiopathic hemolytic anemia, thrombocytopenia, and organ injury [1,2]. Since the initial report in 1924 of a patient with presumed aTTP by Moschcowitz [3] multiple advances have changed the management of this disease. The understanding of the pathophysiology of aTTP came from the observation that unusually large multimers of von Willebrand factor (vWF) were present in patients with TTP [4] which later was found to be due to lack of the vWF–cleaving protease [5,6] subsequently categorized as ADAMTS13 [7]. ADAMTS13 cleaves von Willebrand factor multimers that are secreted from vascular endothelial cells. In the absence of ADAMTS13, the ultra large multimers of vWF cause small-vessel thrombi, platelet consumption and vascular organ damage.

The first major advance in the treatment of aTTP was the use of plasma as a transfusion or therapeutic plasma exchange (TPE) changing the outcome of aTTP from a mostly fatal to a treatable disease [8,9]. Glucocorticoids [8] and rituximab [10,11] to reduce production of the ADAMTS13 inhibitor (autoantibody) have shown benefits and are commonly used.

Caplacizumab is a monoclonal antibody that targets the A1 domain of vWF preventing platelet adhesion to exposed subendothelium at sites of vascular injury [12,13]. In a double-blind controlled trial of patients with aTTP, caplacizumab compared to placebo demonstrated a faster normalization of the platelet count, a lower incidence of complications and a lower rate of recurrence of aTTP [13]. In 2019, the United States Food and Drug Administration approved caplacizumab for the treatment of adult patients with aTTP in combination with plasma exchange and immunosuppressive therapy [14]. Here, we report our single institution experience with caplacizumab since it became commercially available.

## 2. Materials and Methods

This was a retrospective study with the goal of examining our experience with caplacizumab for aTTP in standard practice. We included all patients admitted at our institution that were diagnosed with aTTP and received caplacizumab between the time it became commercially available in the United States of America in 2019 and our data cutoff date of 28 February 2021. There were no exclusion criteria.

We used the definitions for outcomes in aTTP from the International Working Group Consensus in aTTP [15]. Response was defined as a platelet count more than 150,000/uL and LDH less than 1.5 times the upper limit of normal (ULN). Exacerbation meant an aTTP event occurring within 30 days after end of TPE. Complete Response (CR) denoted a response without exacerbation. Relapse represented an aTTP event occurring more than 30 days after the end of TPE. Day 1 was the first day of TPE during the current aTTP event that led to the inclusion into this study and where caplacizumab was used. Event free survival (EFS) was measured from the initiation of TPE and from the start of caplacizumab to the date of relapse or death due to any cause.

## 3. Results

From the time that caplacizumab became commercially available in 2019 until our data cutoff date of February 28, 2020 we identified ten patients that met the inclusion criteria and were included for analyses. Everyone had aTTP and was treated with caplacizumab.

### 3.1. Patients Characteristics

The characteristics of our patients prior to therapy (day 1) are listed in Table 1. Seven were women and three were men with a median age of 52 years (22–78) at the time of caplacizumab administration. Racial demographics demonstrated 5 (50%, *n* = 10) white, 3 (30%, *n* = 10) African American, and 2 (20%, *n* = 10) Hispanic/Latino patients. The median body mass index (BMI) was 34.9 (20.8–56.6) kg/m^2^. Six patients had recurrent aTTP while 4 patients were newly diagnosed at the time of caplacizumab initiation. Two (20%, *n* = 10) patients had a thromboembotic event in the form of a cerebral vascular accident involving the left occipital lobe in one patient and the left frontal lobe in the second patient. Two patients (20%, *n* = 10) had a bleeding event prior to receiving TPE including gross hematuria in one patient and rapid hemoglobin drop following surgical intervention for a thoracic aortic aneurysm in the other subject. Five (50%, *n* = 10) individuals had neurologic symptoms prior to TPE. Of these, 2 subjects reported paresthesia (40%, *n* =5), 3 weakness (60%, *n* = 5), 2 altered mental status (40%, *n* = 5), and 1 blurry vision (20%, *n*=5). None had subjective or objective fever.

The median (range) laboratory values on day 1 demonstrated a platelet count of 29 (9–61) uL, LDH 518 (182–1850) U/L, AST 26 (3–111) U/L ALT 20 (17–33) U/L, bilirubin 1.8 (0.70–4.0) mg/dL and serum creatinine of 1.0 (0.7–2.5) mg/dL. The ADAMTS13 activity and inhibitor were 3 (3–82) % and 1.4 (0.50–32.0) BU, respectively. Only one patient had an elevated troponin prior to TPE at 0.058 ng/mL. All ten patients had a Glasgow Coma Scale of 15 at the time of TPE initiation which was retrospectively calculated based on documented history of presented illness, review of systems, and physical examination.

At the time of TPE discontinuation, the median (range) ADAMTS13 inhibitor was 0.60 (0.50–1.90), and ADAMTS13 activity 42 (3–101) %. The median (range) laboratory values on the day of initiation of caplacizumab revealed a platelet count of 95 (7–314) /uL, LDH 225 (182–562) U/L, AST 20 (11-36) U/L, ALT 19.0 (13–49) U/L, bilirubin 0.7 (0.3–4.7) mg/dL, creatinine 0.9 (0.7–2.5) mg/dL. The median ADAMTS13 inhibitor and activity were 4.30 (1.90–32.0) BU, and 3 (3–82) %, respectively. Every patient was hospitalized. The median duration of hospital stay was 10.5 (4–37) days. Two patients were admitted to the intensive care unit (ICU) for a median length of stay of 6 (3–10) days. 

### 3.2. Treatments

Treatments are summarized in Table 2. TPE was initiated on day 1 concomitantly with corticosteroids at a dose equivalent to prednisone 1 mg/kg or higher. The median number of TPE was 9 (1–33) days and glucocorticoids 24 (5–206) days. All subjects received rituximab starting within the first week of diagnosis, nine at a dose of 375 mg/m^2^ and one at 100 mg/m^2^ weekly for 4 weeks. Two individuals were treated with vincristine 2 mg weekly for 4 weeks for relapsed aTTP with high ADAMTS13 antibody titers. Caplacizumab was initiated at the attending physician’s discretion at a median of 5 (2–34) days after starting TPE and was administered a total of 30 days in 9 (90%, *n* = 10) patients. Discontinuation was at the attending physician’s discretion which occurred after 30 days. Delay in initiating caplacizumab resulted from required insurance prior authorizations and awaiting results of the ADAMTS13 activity. The median number of days on caplacizumab was 31 (8–37). One (10%, *n* = 10) patient was still actively taking caplacizumab. One patient discontinued caplacizumab after 8 days due to normalization of ADAMTS13 activity following the discontinuation of leflunomide [16].

### 3.3. Responses

Responses are summarized in Table 3 and included 6 (60%) CR, 3 (30%) exacerbations and 1 (10%) relapse. Only one patient did not have complete adherence to caplacizumab by missing a few doses, which was the only member of the cohort who relapsed. The median time to normalization of platelet count was 5 (3–50) days, LDH 3 (0–146) days, bilirubin 3 (0–12) days. ADAMTST13 activity normalized with a median of 32.5 (0–89) days.

**Figure 1:**
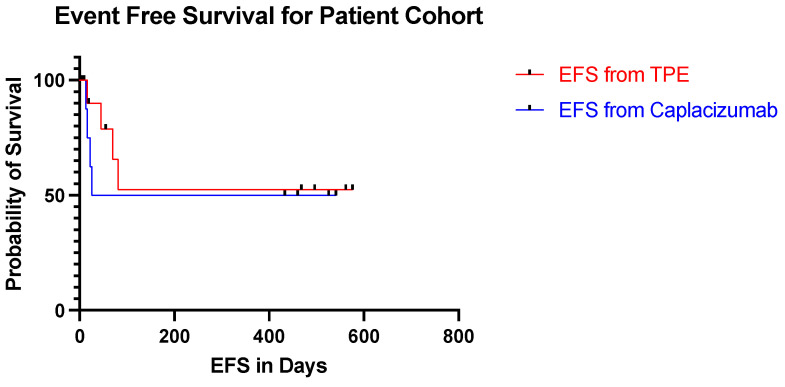
Kaplan–Meier Curves estimates the event free survival (EFS) of patient on caplacizumab from the initiation of therapeutic plasma exchange (TPE) and from the start of caplacizumab.

The median follow up time was 325 days with a range of 16–599 days. The event free survival from day 1 ranged from 16–576 days (Figure 1). Nine patients have discontinued the caplacizumab with one patient still actively taking the medication. In the patient cohort, the event free survival from the discontinuation of caplacizumab ranged from 22 days before stopping caplacizumab to 541 days after stopping caplacizumab. None of the patients suffered from abnormal bleeding, thrombosis or other therapy related toxicities.

## 4. Discussion

Despite advances in the treatment of aTTP with TPE, glucocorticoids and rituximab, morbidity from this condition can be significant. Caplacizumab is a novel anti-vWF agent that has demonstrated efficacy in treating aTTP patients. This study retrospectively examined 10 patients in a standard practice who were diagnosed with aTTP and were treated with caplacizumab. Our patients showed improved outcomes when compared with historical reports prior to the use of caplacizumab [1,8,9,11]. The number of days requiring TPE and glucocorticoid therapy was short at 9 and 24 days, respectively. During our study period, sixty percent of our patients achieved a complete remission and have maintained that response. Thirty percent had exacerbations, and only 1 of the 10 subjects had a relapse after completing therapy. Length of stay in the hospital was improved at a median of 10 days. Only 2 individuals required admission to the ICU for 3 and 10 days each. None of the patients had abnormal bleeding or other adverse events after receiving caplacizumab.

The results from this study are consistent to prior published research. The HERCULES trial randomized 145 patients with aTTP to receive caplacizumab or placebo [13]. The platelet count normalized at 2.69 days following initiation of TPE compared to 5 days in our series. The median number of TPE days was 5 days. Three patients had exacerbations, and 6 patients had relapses [13]. The TITAN trial randomized 75 patients with aTTP to receive caplacizumab or placebo [17]. The time to platelet normalization for the cohort of patients who received TPE prior to randomization to the caplacizumab arm was a median of 2.4 days. The mean number of TPE days was 7.7 days. During the trial period, 3 patients had exacerbations and 19 patients had relapses. Complete remission was achieved in 29 patients [17]. The minimal variation in our findings compared to the results from the HURCULES [13] and TITAN [17] trials are likely due to the controlled environments of clinical trials with rigorous inclusion and exclusion criteria and more selective populations. Within the last few months, three retrospective studies of caplacizumab in treating aTTP were performed in Germany, the United Kingdom (UK) and France [18,19,20]. The results were consistent in all studies. The median duration to platelet normalization was 4–12 days and the median duration of TPE was 5–9 days compared to our finding of 5 and 12 days, respectively. The population of patients in the German, UK, and French studies had a median length of hospital stay of 12–18 days compared to our 10 days. Their median time from initiation of TPE to the time of the first dose of caplacizumab was 2–3 days compared to 5 days in our population [18,19,20].

There are limitations to this study including the number of subjects, retrospective and non-comparative design. While our population size is small compared to the European reports obtained from aggregates of multiple institutions, it is of significant size for a single hospital due to the rarity of the disease. We included all unselected patients that received caplacizumab at our institution to avoid selection or outcomes bias. We acknowledge that a prospective study was not feasible due to caplacizumab being commercially available, and the lack of comparator made a randomized study unviable.

Our results demonstrate real-world experience with a novel drug in a rare condition. Real-world experience is crucial particularly in uncommon diagnoses as they provide data and insight into how therapeutic agents perform in patients when rigorous inclusion and exclusion criteria of clinical trials are absent. Given the nature of retrospective cohort studies, information collected for the purpose of the study are stagnant. In other words, what you see is what you get. While our study had all the critical elements available in the patient’s charts, all the variables were documented by several providers making consistency difficult to maintain. To ensure reliability of the data, we used objective data versus information that was documented in notes as much as possible.

While there are a handful of reports available on patients with aTTP treated with caplacizumab, future research remains imperative. Due to the rarity of aTTP and the unfamiliarity with caplacizumab, there are clinical practices that have not yet implemented this agent into their aTTP treatment regimen, making our experience timely and informative. More research analyzing the safety and efficacy of caplacizumab would be beneficial in establishing its role for the treatment of aTTP.

## 5. Conclusions

In summary, our study provides real-world experience and insight into caplacizumab as a novel, first of its class, anti-vWF agent. Real-world experience in rare conditions such as aTTP is clinically relevant. Our findings confirm caplacizumab in combination with TPE, glucocorticoids and rituximab as a safe and effective therapy for aTTP.

## Figures and Tables

**Table 1:** Patient Cohort Demographics.

Characteristics	Characteristic Breakdown (*N* = 10)	Value Median (Range) Count (%)
Gender	Male	3 (30%)
	Female	7 (70%)
Race	White	5 (50%)
	African American	3 (30%)
	Hispanic/Latino	2 (20%)
Median BMI		34.9 (20.8–56.6)
Thromboembolic or Bleeding Event prior to TPE	Thromboembolic Bleeding	2 (20%)
		2 (20%)
Neurologic Symptoms prior to TPE	Total	5 (50%)
	Weakness	3 (60%, *n* = 5)
	AMS	2 (40%, *n* = 5)
	Numbness	2 (40%, *n* = 5)
	Vision Changes	1 (20%, *n* = 5)
	Glasgow Coma Score	15 (100%, *n* = 10)
Fever Prior to TPE		0 (0%)
aTTP Status at the Time of Caplacizumab Initiation	Initial Recurrent	4 (40%)
		6 (60%)
Baseline Labs before TPE	Platelet (/uL) (*n* = 10)	29 (9–61)
	LDH (U/L) (*n* = 9)	518 (182–1850)
	AST (U/L) (*n* = 9)	26 (3–111)
	ALT (U/L) (*n* = 9)	20 (17–33)
	Bilirubin (mg/dL) (*n* = 9)	1.8 (0.70–4.0)
	Creatinine (mg/dL) (*n* = 10)	1.0 (0.70–2.5)
	Cardiac Troponin (ng/mL) (*n* = 1)	0.058
	ADAMTS13 Inhibitor (BEU) (*n* = 7)	1.40 (0.50–32.0)
	ADAMTS13 Activity (%) (*n* = 9)	3(3–82)
ADAMTS13 Labs after TPE	ADAMTS13 Inhibitor (BEU) (*n* = 4)	0.60 (0.50–1.90)
	ADAMTS13 Activity (%) (*n* = 7)	42 (3–101)
Baseline Labs before Caplacizumab	Platelet (/uL) (*n* = 10)	95 (7–314)
	LDH (U/L) (*n* = 9)	255 (182–562)
	AST (U/L) (*n* = 9)	20 (11–36)
	ALT (U/L) (*n* = 9)	19 (13–49)
	Bilirubin (mg/dL) (*n* = 9)	0.70 (0.30–4.70)
	Creatinine (mg/dL) (*n* = 10)	0.9 (0.70–2.5)
	ADAMTS13 Inhibitor (BEU) (*n* = 7)	4.30 (1.90–32.0)
	ADAMTS13 Activity (%) (*n* = 9)	3 (3–82)
ADAMTS13 Labs at Exacerbation	ADAMTS13 Inhibitor (BEU) (*n* = 1)	1.40
	ADAMTS13 Activity (%) (*n* = 3)	68(3–107)

Abbreviations: BMI: Body Mass Index, aTTP: Acquired Thrombotic Thrombocytopenic Purpura, TPE: Therapeutic Plasma Exchange, AMS: Altered Mental Status, LDH: Lactate Dehydrogensase, AST: Aspartate Aminotransferase, Alanine Aminotranseferase.

**Table 2:** Treatments.

Treatments	Treatment Breakdown	Value Median (Range) Count (%)
Therapeutic Plasma Exchange	Number of Days	12 (1–33)
Glucocorticoid	Number of Days	24 (5–206)
Rituximab	Number of Weeks	2 (1–5)
	Rituximab at 100 mg/m^2^	1 (10%)
	Rituximab at 375 mg/m^2^	9 (90%)
Vincristine	Number of Patients	2 (20%)

**Table 3:** Response Data.

Treatment Response	Response Breakdown	Value Median (Range) Count (%)
Response Criteria	Complete Remission	6 (60%)
	Exacerbation	3 (30%)
	Relapse	1 (10%)
Survival Data from Initiation of TPE in days (*N* = 10)	Event Free Survival	16–576
Hospitalizations and ICU Admissions	Number of Patients Hospitalized	10 (100%)
	Duration of Hospitalization in Days	10 (4–37)
	Number of Patients with an ICU Stay	2 (20%)
	Duration of ICU Stay in Days	6 (3–10)
Adverse Events while on Caplacizumab	Thrombotic Event	0 (0%)
	Bleeding Event	0 (0%)
	Other	0 (0%)
Reason for Drug Discontinuation (*n* = 8)	Completion of Prescribed Course Patients still on Caplacizumab (*n* = 10)	9 (90%)
		1 (10%)

Abbreviations: TPE: Therapeutic Plasma Exchange; ICU: Intensive Care Unit.

## Data Availability

The data presented in this study are available in the tables of this article.
